# Supplementary material for: A Systematic Review of Joint Spatial and Spatiotemporal Models in Health Research
Source: Int J Environ Res Public Health. 2023 Mar 28;20(7):5295. doi: 10.3390/ijerph20075295 (PMC10094468; doi:10.3390/ijerph20075295)
Supplement: Supplementary file 1 [file ijerph-20-05295-s001.zip › ijerph-2195131-supplementary.pdf]

Table S1: Databases and searching terms

Databases	Search terms
**Medline**	(Multivariate spatiotemporal [MeSH Terms] OR Bivariate spatiotemporal [MeSH Terms] OR Multivariate spatio-temporal [MeSH Terms] OR Bivariate spatio-temporal [MeSH Terms] OR Joint shared spatial model [MeSH Terms] OR Joint space-time model*[MeSH Terms] OR Multivariate space-time model*[MeSH Terms] OR Bivariate space-time model [MeSH Terms] OR (Small area analys* AND Shared component model[MeSH Terms]) OR (Disease mapping AND Shared component model[MeSH Terms]) OR Space-time mixture model [MeSH Terms] OR Shared component Model [MeSH Terms] OR (Spatial analys* AND Joint model*[MeSH Terms]) OR Joint spatial model*[MeSH Terms] OR Joint spatial analys*[MeSH Terms] OR Shared latent component model[MeSH Terms] OR Joint model AND Spatial model [MeSH Terms] OR Spatial factor analys*[MeSH Terms] OR (Risk map* and Shared component model*[MeSH Terms]) OR Shared spatial model*[MeSH Terms] OR Multivariate spatial analys*[MeSH Terms] OR Bivariate spatial analys*[MeSH Terms] OR Bivariate conditional autoregressive model[MeSH Terms] OR Multivariate conditional autoregressive model[MeSH Terms] OR Joint conditional autoregressive model[MeSH Terms] OR Joint spatial autocorrelation[MeSH Terms] OR Bivariate spatial autocorrelation[MeSH Terms] OR Multivariate spatial autocorrelation [MeSH Terms] OR Spatial co-cluster*[MeSH Terms] OR spatio-temporal co-cluster*[MeSH Terms]) AND (("2011/01/01"[Date - Publication] : "2022/09/14"[Date - Publication]))
**Embase**	(Multivariate spatiotemporal.mp OR Bivariate spatiotemporal.mp OR Multivariate spatio-temporal.mp OR Bivariate spatio-temporal.mp OR Joint shared spatial model.mp OR Joint space-time model*.mp OR Multivariate space-time model*.mp OR Bivariate space-time model.mp OR (Small area analys*.mp AND Shared component model.mp) OR (Disease mapping AND Shared component model.mp) OR Space-time mixture model.mp OR Shared component Model.mp OR (Spatial analys*.mp AND Joint model*.mp) OR Joint spatial model*.mp OR Joint spatial analys*.mp OR Shared latent component model.mp OR (Joint model*.mp AND Spatial model*.mp) OR Spatial factor analys*.mp OR (Risk map*.mp and Shared component model*.mp) OR Shared spatial model*.mp OR Multivariate spatial analys*.mp OR Bivariate spatial analys*.mp OR Bivariate conditional autoregressive model.mp OR Multivariate conditional autoregressive model.mp OR Joint conditional autoregressive model.mp OR Joint spatial autocorrelation.mp OR Bivariate spatial autocorrelation.mp OR Multivariate spatial autocorrelation.mp OR Spatial co-cluster*.mp OR spatio-temporal co-cluster*.mp AND ("2011/01/01"[Date - Publication] : "2022/09/14"[Date - Publication]))
**PschINFO**	(Multivariate spatiotemporal.mp OR Bivariate spatiotemporal.mp OR Multivariate spatio-temporal.mp OR Bivariate spatio-temporal.mp OR Joint shared spatial model.mp OR Joint space-time model*.mp OR Multivariate space-time model*.mp OR Bivariate space-time model.mp OR

	(Small area analys*.mp AND Shared component model.mp) OR (Disease mapping AND Shared component model.mp) OR Space-time mixture model.mp OR Shared component Model.mp OR (Spatial analys*.mp AND Joint model*.mp) OR Joint spatial model*.mp OR Joint spatial analys*.mp OR Shared latent component model.mp OR (Joint model*.mp AND Spatial model*.mp) OR Spatial factor analys*.mp OR (Risk map*.mp and Shared component model*.mp) OR Shared spatial model*.mp OR Multivariate spatial analys*.mp OR Bivariate spatial analys*.mp OR Bivariate conditional autoregressive model.mp OR Multivariate conditional autoregressive model.mp OR Joint conditional autoregressive model.mp OR Joint spatial autocorrelation.mp OR Bivariate spatial autocorrelation.mp OR Multivariate spatial autocorrelation.mp OR Spatial co-cluster*.mp OR spatio-temporal co-cluster*.mp AND ("2011/01/01"[Date - Publication] : "2022/09/14"[Date - Publication]))
**PubMed**	((((((((((((((((((((((((((((Multivariate spatiotemporal[MeSH Terms]) OR (Bivariate spatiotemporal[MeSH Terms])) OR (Multivariate spatio-temporal[MeSH Terms])) OR (Bivariate spatio-temporal[MeSH Terms])) OR (Joint shared spatial model[MeSH Terms])) OR (Joint space-time model*[MeSH Terms])) OR (Multivariate space-time model*[MeSH Terms])) OR (Bivariate space-time model[MeSH Terms])) OR (Small area analys* AND Shared component model[MeSH Terms])) OR (Disease mapping AND Shared component model[MeSH Terms])) OR (Space-time mixture model[MeSH Terms])) OR (Shared component Model[MeSH Terms])) OR (Spatial analys* AND Joint model*[MeSH Terms])) OR (Joint spatial model*[MeSH Terms])) OR (Joint spatial analys*[MeSH Terms])) OR (Shared latent component model[MeSH Terms])) OR (Joint model AND Spatial model[MeSH Terms])) OR (Spatial factor analys*[MeSH Terms])) OR (Risk map* and Shared component model*[MeSH Terms])) OR (Shared spatial model*[MeSH Terms])) OR (Multivariate spatial analys*[MeSH Terms])) OR (Bivariate spatial analys*[MeSH Terms])) OR (Bivariate conditional autoregressive model[MeSH Terms])) OR (Multivariate conditional autoregressive model[MeSH Terms])) OR (Joint conditional autoregressive model[MeSH Terms])) OR (Joint spatial autocorrelation[MeSH Terms])) OR (Bivariate spatial autocorrelation[MeSH Terms])) OR (Multivariate spatila autocorrelation[MeSH Terms])) OR (Spatial co-cluster*[MeSH Terms])) OR (spatio-temporal co-cluster*[MeSH Terms])) AND (("2011/01/01"[Date - Publication] : "2022/09/14"[Date - Publication]))
**SCOPUS**	TITLE-ABS-KEY (Multivariate spatiotemporal) OR TITLE-ABS-KEY (Bivariate spatiotemporal) OR TITLE-ABS-KEY(Multivariate spatio-temporal) OR TITLE-ABS-KEY (Bivariate spatio-temporal) OR TITLE-ABS-KEY (Joint shared spatial model) OR TITLE-ABS-KEY (Joint space-time model*) OR TITLE-ABS-KEY (Multivariate space-time model*) OR TITLE-ABS-KEY (Bivariate space-time model) OR (TITLE-ABS-KEY(Small area analys*) AND TITLE-ABS-KEY (Shared component model)) OR (TITLE-ABS-KEY (Disease mapping) AND TITLE-ABS-KEY (Shared component model)) OR TITLE-ABS-KEY (Space-time mixture model) OR TITLE-ABS-

	KEY (Shared component Model) OR (TITLE-ABS-KEY (Spatial analys*) AND Joint model*)) OR TITLE-ABS-KEY (Joint spatial model*) OR TITLE-ABS-KEY (Joint spatial analys*) OR TITLE-ABS-KEY (Shared latent component model) OR (TITLE-ABS-KEY (Joint model*) AND Spatial model*) OR TITLE-ABS-KEY (Spatial factor analys*) OR (TITLE-ABS-KEY (Risk map*) and TITLE-ABS-KEY (Shared component model*)) OR TITLE-ABS-KEY (Shared spatial model*) OR TITLE-ABS-KEY (Multivariate spatial analys*) OR TITLE-ABS-KEY (Bivariate spatial analys*) OR TITLE-ABS-KEY (Bivariate conditional autoregressive model) OR TITLE-ABS-KEY (Multivariate conditional autoregressive model) OR TITLE-ABS-KEY (Joint conditional autoregressive model) OR TITLE-ABS-KEY (Joint spatial autocorrelation) OR TITLE-ABS-KEY (Bivariate spatial autocorrelation) OR TITLE-ABS-KEY (Multivariate spatial autocorrelation) OR TITLE-ABS-KEY (Spatial co-cluster*) OR TITLE-ABS-KEY (spatio-temporal co-cluster*) AND ("2011/01/01"[Date - Publication] : "2022/09/14"[Date - Publication]))
**Emcare**	(Multivariate spatiotemporal.mp OR Bivariate spatiotemporal.mp OR Multivariate spatio-temporal.mp OR Bivariate spatio-temporal.mp OR Joint shared spatial model.mp OR Joint space-time model*.mp OR Multivariate space-time model*.mp OR Bivariate space-time model.mp OR (Small area analys*.mp AND Shared component model.mp) OR (Disease mapping AND Shared component model.mp) OR Space-time mixture model.mp OR Shared component Model.mp OR (Spatial analys*.mp AND Joint model*.mp) OR Joint spatial model*.mp OR Joint spatial analys*.mp OR Shared latent component model.mp OR (Joint model*.mp AND Spatial model*.mp) OR Spatial factor analys*.mp OR (Risk map*.mp and Shared component model*.mp) OR Shared spatial model*.mp OR Multivariate spatial analys*.mp OR Bivariate spatial analys*.mp OR Bivariate conditional autoregressive model.mp OR Multivariate conditional autoregressive model.mp OR Joint conditional autoregressive model.mp OR Joint spatial autocorrelation.mp OR Bivariate spatial autocorrelation.mp OR Multivariate spatial autocorrelation.mp OR Spatial co-cluster*.mp OR spatio-temporal co-cluster*.mp AND ("2011/01/01"[Date - Publication] : "2022/09/14"[Date - Publication]))

Table S2: Risk of bias tool for assessment

	Criterion[1]	Considerations[1]	Score considerations (0, none, 1, poor, 2, good)	
	**(A) Screening questions**			Definition, Max 4 points
1	Does the paper clearly address aims and objectives?	Is the paper relevant to the objectives of the systematic review of Bayesian modelling?	0 not stated<br>1 stated but vague<br>2 stated and focussed	
2	Is the setting and population clearly defined?	Does the paper clearly state the setting (e.g. number of geographical location, number of dengue cases)?	0 not stated<br>1 stated but vague<br>2 stated and focussed	
	**(B) Assessed the validity of model**			
3	Is the model structure clearly described and appropriate for the research question?	Is there a description of model structure (prior for space, time or space-time)?<br>Does the model structure include covariates?	0 not appropriate model structure, or no description of model<br>1 incomplete description<br>2 complete description	Model methods, Max 4 points
4	Are the modelling methods appropriate for the research question?	Were the modelling methods clearly described, and suited to the research question?	0 not appropriate modelling method, or no description of method<br>1 incomplete description<br>2 complete description	
5	Are the parameters, ranges and data source specified?	Are all parameters and their ranges reported?<br>Are the data sources for parameters reported?	0 poorly reported<br>1 some information missing<br>2 complete reporting of parameters, ranges and data sources	Model inputs, Max 4 points
6	Is the quality of data considered?	Are data limitations discussed?	0 no sources of uncertainty<br>1 partially addressed, and/or data inappropriate<br>2 fully addressed	
	**(C) Assessed the overall results and study conclusion**			
7	Have the results been clearly and completely presented?	Do the results match the aims and objectives?	0 not reported, very unclear<br>1 stated, but not directly aligned	Results, Max 4 points

			with research question<br>2 valuable and aligned with research question	
8	Are the results appropriately interpreted and discussed in context?	Are the results of the study discussed in context and generalisability considered?	0 no discussion<br>1 some discussion but key points and/or limitations missed<br>2 full discussion of key points, limitations discussed	

Total score, Max 16
Very high > 13
High 11-13
Medium 8 -10
Low < 8

Table S3: List of publications included for systematic review

ID	Included studies (references)
1	Adegboye OA, Al-Saghir M, LEUNG DH. Joint spatial time-series epidemiological analysis of malaria and cutaneous leishmaniasis infection. Epidemiology & Infection. 2017 Mar;145(4):685-700
2	Adeyemi RA, Zewotir T, Ramroop S. Joint spatial mapping of childhood anemia and malnutrition in sub-Saharan Africa: a cross-sectional study of small-scale geographical disparities. African health sciences. 2019 Nov 7;19(3):2692-712
3	Ahmadipanahmehrabadi V, Hassanzadeh A, Mahaki B. Bivariate spatio-temporal shared component modeling: Mapping of relative death risk due to colorectal and stomach cancers in Iran provinces. International Journal of Preventive Medicine. 2019;10
4	Baker J, White N, Mengersen K, Rolfe M, Morgan GG. Joint modelling of potentially avoidable hospitalisation for five diseases accounting for spatiotemporal effects: A case study in New South Wales, Australia. PLoS One. 2017 Aug 30;12(8):e0183653
5	Bermudi PM, Pellini AC, Rebolledo EA, Diniz CS, Aguiar BS, Ribeiro AG, Failla MA, Baquero OS, Chiaravalloti-Neto F. Spatial pattern of mortality from breast and cervical cancer in the city of São Paulo. Revista de Saúde Pública. 2020 Dec 16;54
6	Besharati MM, Kashani AT, Li Z, Washington S, Prato CG. A bivariate random effects spatial model of traffic fatalities and injuries across Provinces of Iran. Accident Analysis & Prevention. 2020 Mar 1;136:105394.
7	Carabali M, Schmidt AM, Restrepo BN, Kaufman JS. A joint spatial marked point process model for dengue and severe dengue in Medellin, Colombia. Spatial and Spatio-temporal Epidemiology. 2022 Jun 1;41:100495
8	Carroll R, Lawson AB, Faes C, Kirby RS, Aregay M, Watjou K. Extensions to multivariate space time mixture modeling of small area cancer data. International journal of environmental research and public health. 2017 May;14(5):503.
9	Chamanpara P, Moghimbeigi A, Faradmal J, Poorolajal J. Joint disease mapping of two digestive cancers in Golestan Province, Iran using a shared component model. Osong public health and research perspectives. 2015 Jun 1;6(3):205-10
10	Chidumwa G, Maposa I, Kowal P, Micklesfield LK, Ware LJ. Bivariate joint spatial modeling to identify shared risk patterns of hypertension and diabetes in south africa: Evidence from WHO SAGE South Africa Wave 2. International journal of environmental research and public health. 2021 Jan;18(1):359
11	Cramb SM, Baade PD, White NM, Ryan LM, Mengersen KL. Inferring lung cancer risk factor patterns through joint Bayesian spatio-temporal analysis. Cancer Epidemiology. 2015 Jun 1;39(3):430-9
12	Darikwa TB, Manda S, Lesaoana M. Assessing joint spatial autocorrelations between mortality rates due to cardiovascular conditions in South Africa. Geospatial Health. 2019 Nov 6;14(2)
13	Darikwa TB, Manda S, Lesaoana M. Assessing joint spatial autocorrelations between mortality rates due to cardiovascular conditions in South Africa. Geospatial Health. 2019 Nov 6;14(2)
14	Desjardins MR, Whiteman A, Casas I, Delmelle E. Space-time clusters and co-occurrence of chikungunya and dengue fever in Colombia from 2015 to 2016. Acta tropica. 2018 Sep 1;185:77-85
15	Freitas LP, Carabali M, Yuan M, Jaramillo-Ramirez GI, Balaguera CG, Restrepo BN, Zinszer K. Spatio-temporal clusters and patterns of spread of dengue, chikungunya, and Zika in Colombia. medRxiv. 2022 Jan 1
16	Kazembe LN, Kandala NB. Estimating areas of common risk in low birth weight and infant mortality in Namibia: A joint spatial analysis at sub-regional level. Spatial and spatio-temporal Epidemiology. 2015 Jan 1;12:27-37

17 Kinyoki DK, Manda SO, Moloney GM, Odundo EO, Berkley JA, Noor AM, Kandala NB. Modelling the ecological comorbidity of acute respiratory infection, diarrhoea and stunting among children under the age of 5 years in Somalia. International Statistical Review. 2017 Apr;85(1):164-76

18 Kline D, Hepler S, Bonny A, McKnight E. A joint spatial model of opioid-associated deaths and treatment admissions in Ohio. Annals of epidemiology. 2019 May 1;33:19-23.

19 Kramer MR, Williamson R. Multivariate Bayesian spatial model of preterm birth and cardiovascular disease among Georgia women: Evidence for life course social determinants of health. Spatial and Spatio-temporal Epidemiology. 2013 Sep 1;6:25-35.

20 Law J, Perlman C. Exploring geographic variation of mental health risk and service utilization of doctors and hospitals in Toronto: A shared component spatial modeling approach. International journal of environmental research and public health. 2018 Apr;15(4):593

21 Lawson AB, Carroll R, Castro M. Joint spatial Bayesian modeling for studies combining longitudinal and cross-sectional data. Statistical Methods in Medical Research. 2014 Dec;23(6):611-24.

22 Lawson A, Schritz A, Villarroel L, Aguayo GA. Multi-Scale Multivariate Models for Small Area Health Survey Data: A Chilean Example. International journal of environmental research and public health. 2020 Mar;17(5):1682.

23 Mahaki B, Mehrabi Y, Kavousi A, Akbari ME, Waldhoer T, Schmid VJ, Yaseri M. Multivariate disease mapping of seven prevalent cancers in Iran using a shared component model. Asian Pac J Cancer Prev. 2011 Jan 1;12(9):2353-8.

24 Mahaki B, Mehrabi Y, Kavousi A, Schmid VJ. Joint spatio-temporal shared component model with an application in Iran Cancer Data. Asian Pacific journal of cancer prevention: APJCP. 2018;19(6):1553

25 Mahaki B, Mehrabi Y, Kavousi A, Schmid VJ. Joint spatio-temporal shared component model with an application in Iran Cancer Data. Asian Pacific journal of cancer prevention: APJCP. 2018;19(6):1553

26 Neelon B, Anthopolos R, Miranda ML. A spatial bivariate probit model for correlated binary data with application to adverse birth outcomes. Statistical methods in medical research. 2014 Apr;23(2):119-33.

27 Odhiambo JN, Sartorius B. Joint spatio-temporal modelling of adverse pregnancy outcomes sharing common risk factors at sub-county level in Kenya, 2016–2019. BMC public health. 2021 Dec;21(1):1-3

28 Okango E, Mwambi H, Ngesa O, Achia T. Semi-parametric spatial joint modeling of HIV and HSV-2 among women in Kenya. PloS one. 2015 Aug 10;10(8):e0135212.

29 Orunmoluyi OS, Gayawan E, Manda S. Spatial Co-Morbidity of Childhood Acute Respiratory Infection, Diarrhoea and Stunting in Nigeria. International Journal of Environmental Research and Public Health. 2022 Feb 6;19(3):1838.

30 Otiende VA, Achia TN, Mwambi HG. Bayesian hierarchical modeling of joint spatiotemporal risk patterns for Human Immunodeficiency Virus (HIV) and Tuberculosis (TB) in Kenya. PloS one. 2020 Jul 2;15(7):e0234456.

31 Raei M, Schmid VJ, Mahaki B. Bivariate spatiotemporal disease mapping of cancer of the breast and cervix uteri among Iranian women. Geospatial Health. 2018 May 8;13(1).

32 Ransome Y, Subramanian SV, Duncan DT, Vlahov D, Warren J. Multivariate spatiotemporal modeling of drug-and alcohol-poisoning deaths in New York City, 2009–2014. Spatial and spatio-temporal epidemiology. 2020 Feb 1;32:100306.

33 Roberts DJ, Zewotir T. Copula geoadditive modelling of anaemia and malaria in young children in Kenya, Malawi, Tanzania and Uganda. Journal of Health, Population and Nutrition. 2020 Dec;39(1):1-4.

34 Schur N, Gosoniu L, Raso G, Utzinger J, Vounatsou P. Modelling the geographical distribution of co-infection risk from single-disease surveys. Statistics in medicine. 2011 Jun 30;30(14):1761-76.

35 Stensgaard AS, Vounatsou P, Onapa AW, Simonsen PE, Pedersen EM, Rahbek C, Kristensen TK. Bayesian geostatistical modelling of malaria and lymphatic filariasis infections in Uganda: predictors of risk and geographical patterns of co-endemicity. Malaria journal. 2011 Dec;10(1):1-5.

36 Stoppa G, Mensi C, Fazzo L, Minelli G, Manno V, Consonni D, Biggeri A, Catelan D. Spatial analysis of shared risk factors between pleural and ovarian cancer mortality in Lombardy (Italy). International journal of environmental research and public health. 2022 Mar 15;19(6):3467.

37 Norwood TA, Encisa C, Wang X, Seliske L, Cunningham J, De P. A Bayesian shared components modeling approach to develop small area indicators of social determinants of health with measures of uncertainty. Canadian Journal of Public Health. 2020 Jun;111(3):342-57.

38 Adebayo SB, Gayawan E, Heumann C, Seiler C. Joint modeling of Anaemia and Malaria in children under five in Nigeria. Spatial and spatio-temporal epidemiology. 2016 May 1;17:105-15.

39 Asmarian N, Ayatollahi SM, Sharafi Z, Zare N. Bayesian spatial joint model for disease mapping of zero-inflated data with R-INLA: A simulation study and an application to male breast cancer in Iran. International Journal of Environmental Research and Public Health. 2019 Nov;16(22):4460.

40 Huang G, Lee D, Scott EM. Multivariate space-time modelling of multiple air pollutants and their health effects accounting for exposure uncertainty. Statistics in medicine. 2018 Mar 30;37(7):1134-48

41 Kang J, Zhang N, Shi R. A Bayesian nonparametric model for spatially distributed multivariate binary data with application to a multidrug-resistant tuberculosis (MDR-TB) study. Biometrics. 2014 Dec;70(4):981-92.

42 Law J, Quick M, Jadavji A. A Bayesian spatial shared component model for identifying crime-general and crime-specific hotspots. Annals of GIS. 2020 Jan 2;26(1):65-79

43 Roberts DJ, Zewotir T. Shared component modelling of early childhood anaemia and malaria in Kenya, Malawi, Tanzania and Uganda. BMC Pediatrics. 2022 Dec;22(1):1-1.
